# Practice makes the deficiency of global motion detection in people with pattern-related visual stress more apparent

**DOI:** 10.1371/journal.pone.0193215

**Published:** 2018-02-15

**Authors:** Ding Han, Jana Wegrzyn, Hua Bi, Ruihua Wei, Bin Zhang, Xiaorong Li

**Affiliations:** 1 School of Optometry and Ophthalmology, Tianjin Medical University Eye Hospital, Tianjin, China; 2 College of Optometry, Nova Southeastern University, Fort Lauderdale, Florida, United States of America; University of Nevada Reno, UNITED STATES

## Abstract

**Aims:**

Pattern-related visual stress (PRVS) refers to the perceptual difficulties experienced by some individuals when exposed to high contrast striped patterns. People with PRVS were reported to have reduced sensitivity to global motion at baseline testing and the difference disappears at a second estimate. The present study was to investigate the effect of practice on global motion threshold in adults with and without PRVS.

**Methods:**

A total of 101 subjects were recruited and the Wilkins & Evans Pattern Glare Test was used to determine if a subject had PRVS. The threshold to detect global motion was measured with a random dot kinematogram. Each subject was measured 5 times at the first visit and again a month later. Receiver operating characteristic (ROC) curve analysis was applied to show the agreement between the two tests.

**Results:**

Twenty-nine subjects were classified as having PRVS and 72 were classified as normal. At baseline, the threshold to detect global motion was significantly higher in subjects with PRVS (0.832 ± 0.098 vs. 0.618 ± 0.228, p < 0.001). After 5 sessions, the difference between the normal and subjects with PRVS increased (0.767 ± 0.170 vs. 0.291 ± 0.149, p < 0.001). In ROC analysis, the area under the curve (AUC) improved from 0.792 at baseline to 0.964 at the fifth session. After a one-month break, the difference between normal and subjects with PRVS was still significant (0.843 ± 0.169 vs. 0.407 ± 0.216, p < 0.001) and the AUC was 0.875.

**Conclusion:**

The ability to detect global motion is impaired in persons with PRVS and the difference increased after additional sessions of practice.

## Introduction

Pattern-related visual stress (PRVS) refers to the discomfort experienced by some people while viewing high contrast and repetitive patterns. This includes bodily symptoms, such as malaise and nausea, and perceptual symptoms ranging from illusions of color to seeing patterns vibrating [1]. Patterns with large deviations from natural image statistics, such as a high contrast striped grating in the range of three to four cycles per degree, often result in peak PRVS and anomalous experiences [2–5]. Studies suggest the effects of PRVS may be significant in daily life in healthy non-clinical individuals [6]. Those affected show less accuracy in identifying words versus non-words and are slower in visual searches, potentially affecting reading performance [7]. Because the population’s use of computers during daily activity is increasing, the consequences of high contrast images, motion, and repetitive tasks among people with PRVS need additional studies.

PRVS is a unique set of symptoms that should not be confused with blur or fatigue. Ocular factors, such as instability in fixation and increased microfluctuation in accommodation, are unlikely to be explanations for the phenomenon [8–11]. The neural mechanism underlying PRVS is generally thought to be of cortical origin. The fact that PRVS is more likely to be evoked under binocular than monocular conditions also supports this view [12]. Unlike the natural images that cause a sparse response in the visual system, visually averse stimuli may cause an anomalous response as found in PRVS, as a result of either cortical hyperexcitability or poor cortical inhibition [5, 13–16]. Previous studies suggested an overload in extrastriate dorsal visual pathway in PRVS [17].

Migraine has a strong correlation to pattern glare with 82% of migraineurs exhibiting PRVS [18–20]. The visual stimuli that trigger migraine and PRVS share common features [21]. Those with statistical properties away from the natural scenes tend to evoke both migraine and PRVS [22–24]. Before the onset of the headache, up to 24 hours before, migraineurs’ susceptibility to pattern glare is increased [25]. The distortions perceived by migraineurs tend to be on the same side of aura [26, 27]. While the headaches are usually unilateral, the distortions tend to be predominant in one visual hemifield [5, 25]. Moreover, cortical hyperexcitability has also been reported in the visual cortex of the people with migraines [28, 29]. Therefore, PRVS and migraine should be viewed as the two ends of a continuum, with PRVS in the non-clinical population who experience abnormal illusions and migraine in the clinical population who suffer migraine attacks [16].

People with migraine do not perform well in motion coherence task [28, 30, 31], in which one needs to detect the elements moving coherently (the same direction) from the elements moving at random directions over a large space. Such a function could not be achieved in the primary visual cortex (V1) since the neurons there have small receptive fields and are only capable of analyzing location motion (movements in a small spatial region) [32]. The direction of the coherent motion could not be determined from tracking the trajectory of a single element. The outputs from many V1 neurons need to be pooled and integrated to extract the global motion information [33]. This step is done by the neurons in the medial temporal (MT) and medial superior temporal (MST) areas, where the neurons have much larger receptive fields and suitable for global motion analysis. The impaired performance in migraine is considered associated with the cortical hyperexcitability [28, 30, 31].

The global motion in people with PRVS has been less studied, possibly due the fact many of them are healthy nonclinical persons. The findings from the few existing studies are sketchy and even contradicting to each other. In one study, Simmers reported that the thresholds to detect global motion in people with PRVS are not significantly different from that in the normal population [34]. In another study, impaired thresholds for global motion detection are found in people with PRVS when tested for the first time. However, an improvement after a second attempt usually makes the difference insignificant [35]. Considering the close correlation between PRVS and migraine, such as common triggering stimuli and cortical hyperexcitability [5, 13–16], the global motion in PRVS deserves a closer examination.

In these previous studies, whether a subject has PRVS was assessed with different approaches. One is based on the subjects’ memory of symptoms that have occurred during daily activity, particularly those related to readings [35]. The other approach is based on observation whether a subject showed certain signs, such as voluntarily wearing colored filters over a sustained period of time [34]. In this study, we used the Wilkins & Evans Pattern Glare Test (PGT), in which a pattern likely to induce PRVS is presented to the subjects who report the occurrence of the visual disturbance just experienced [26]. Comparing to previous methods, PGT has several advantages. First, it is independent of the process of choosing a colour overlay. Second, it collects the subjects’ immediate symptoms after viewing the patterns, instead of recalling the symptoms encountered in the past. Third, the normative values for the normal population and specific diagnostic criteria have been clearly established by the researchers who invented this test.

Therefore, in this study, we used the PGT to identify the subjects with PRVS first. Then we investigated if the subject PRVS have significantly worse performance in detecting global motion compared to the people without PRVS. Moreover, we tested whether practice can alleviate such impairment.

## Materials and methods

### Subjects

A total of 101 unselected university students (27 male vs. 74 female, aged 19–35) participated in the study. The average spherical equivalent (SE) for all the eyes were -2.59 ± 2.43 D. The average SE was -2.67 ± 2.47 D for the right eyes and -2.50 ± 2.45 D for left eyes. There was no significant difference between the SE of the two eyes (p = 0.61, ranksum test). No information about the prevalence of specific learning difficulties, migraine, epilepsy, and medications was collected. All subjects were informed about the details of the study and written consent was obtained. This study protocol was approved by the Institutional Review Board of the Nova Southeastern University.

### Pattern glare test

Whether a subject has PRVS was determined with the Wilkins & Evans Pattern Glare Test [36]. In short, a field of horizontal stripes of low (0.5 cycles per degree), middle (3 cpd), and high (12 cpd) spatial frequencies (SF) was displayed. A grating with a middle range SF (3 cpd) served as the main test, which is expected to elicit the most visual discomfort. In addition, the pattern glare test had two more control gratings. The one with the lowest frequency (0.5 cpd) was designed to filter out the subjects who would be highly suggestible and report many visual distortions even when they perceived none. The one with a high frequency (12 cpd) was designed to filter out the distortions caused by optical reasons. The subjects were asked to keep their fixation on a dot at the center of the grating for about 5 seconds, and then record any distortions seen on the record sheet. A subject with a score of > 3 on the middle SF pattern or a score of > 1 on the difference between mid and high SF patterns (mid—high) was defined as having PRVS [26].

### Global motion test

**Fig 1:**
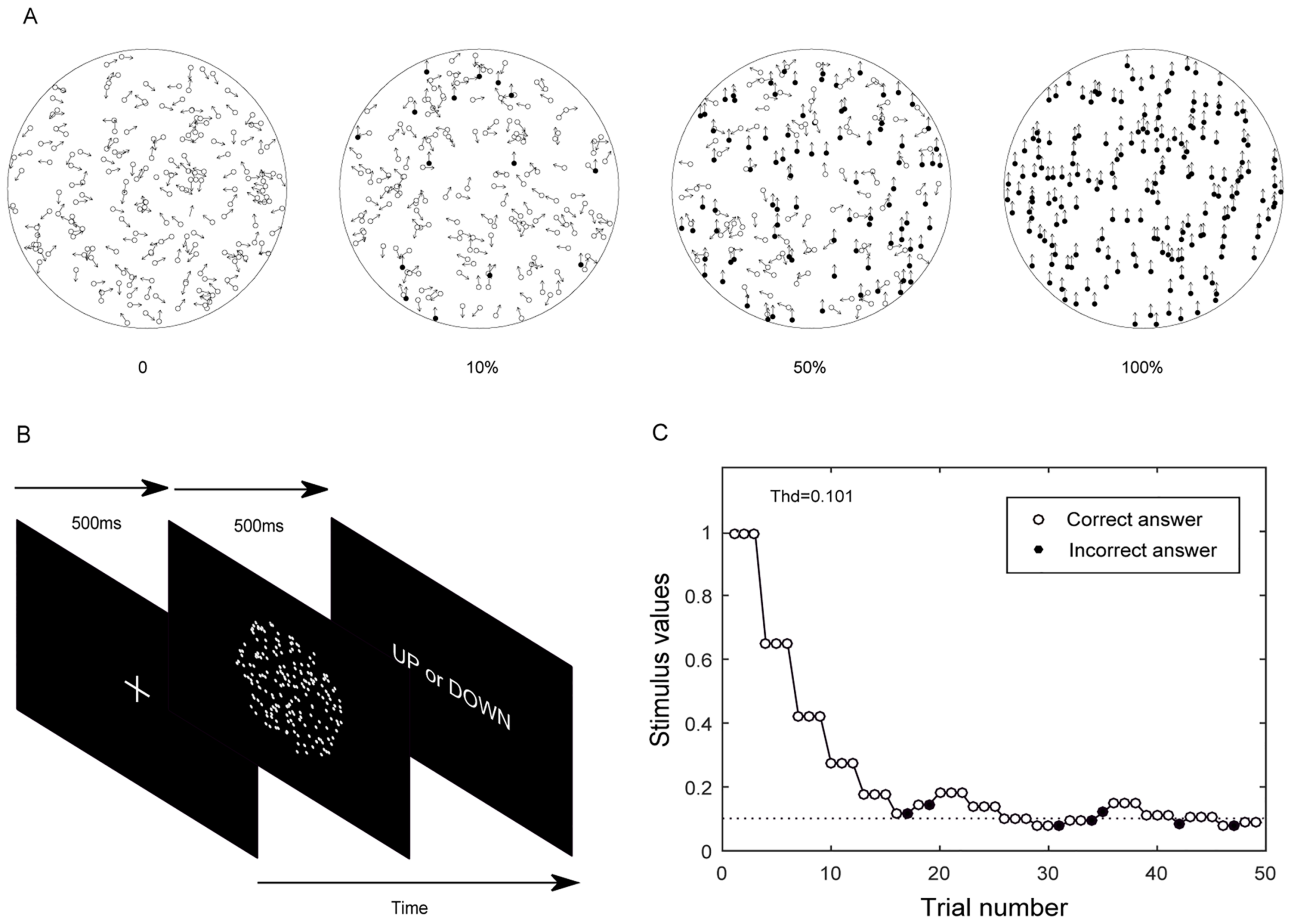
Stimulus and experimental procedure. (A) Kinematogram with different levels of coherence with dots moving in the same direction presented as filled one. (B) Experimental procedure. (C) An example of results obtained from a staircase with a 3-down-1-up paradigm.

The threshold to detect global motion was measured with a random dot kinematogram. A total of 200 white dots, each with high luminance (80 cd/m^2^), were presented on a low luminance (0.3 cd/m^2^) background. The display was calibrated with a luminance meter (LS-100; Konica Minolta, Osaka, Japan). The stimulus was viewed binocularly from a head and chin rest. All stimuli was generated in Matlab (version 2012a, The MathWorks Inc., Natick, MA) using Psychophysics Toolbox and displayed on a BenQ LCD 27-in monitor (BenQ Corporation, Taipei, Taiwan) with a resolution of 2560 × 1440 pixels and a 60 Hz refresh rate [37, 38]. The tests were performed at a distance of 60 cm with a display size of 53 × 31 degree. The moving dots were presented within a 12 degree circular window and consisted of two kinds. The signal dots moved coherently towards the same direction, while the noise ones moved in random directions with an angular velocity of 5.0 deg/s collectively. A single dot size subtended 0.16 degree, with each dot having a lifetime of 200 ms (12 movie frames), after which the dot disappeared and was then regenerated at a random location within the circular window. The duration of each trial was 500 ms (Fig 1).

Observers were asked to identify the direction of the perceived global motion, i.e. up vs. down, in a single-interval identification paradigm. An experimental trial consisted of the following sequence: (1) A white fixation cross appeared on the screen, (2) the fixation cross disappeared and the stimulus was presented for 500 ms; (3) a text prompt appeared until the subject responded by pressing one of two keys on a keypad, up or down; and (4) the text disappeared and audio feedback was provided to indicate the completion of a trial. The coherence of the moving dots, that is the percentage of signal dots, was adjusted according to a 3-down-1-up staircase with a beginning coherence of 100%. The threshold was estimated from the arithmetic mean of the last 6 reversals with 8 reversals in total per test. The test was repeated 5 times continuously, and was repeated once again after a 1-month interval.

One person collected all the data of pattern glare test and another person collected global motion test results. Those two persons were masked from each other. The participants were also masked.

### The agreement between pattern glare test and global motion

All statistical analyses were performed using SPSS statistical package 19 (SPSS, IBM, Chicago, IL, USA). To compare the threshold to global motions, which did not follow a normal distribution as confirmed with the Kolmogorov-Smirnov test, a Mann-Whitney U test was used. A receiver operating characteristic (ROC) curve was employed to evaluate the agreement between the two tests at baseline, after 5 sessions of training, and after the 1-month break. The area-under-the-curve (AUC) was used as the index to reflect the goodness of the agreement. Statistical significance was defined as p < 0.05.

## Results

### Pattern glare data

Based on the criteria, 72 subjects were classified as normal without PRVS and other 29 were classified as subjects who experienced PRVS. Their scores are summarized in Table 1. G1 to G3 presents the average number of visual illusion experienced while viewing the gratings with low, middle, and high SF respectively. G2-G3 indicates the difference in numbers of illusion experienced viewing gratings of middle SF versus grating of high SF.

**Table 1:** Scores on the pattern glare test.

Score	Normal	PRVS	P value
G1	0.514 ± 0.769	0.828 ± 0.889	0.079
G2	1.723 ± 1.077	3.862 ± 1.093	< 0.001
G3	2.111 ± 1.295	2.828 ± 1.891	0.031
G2-G3	-0.389 ± 1.082	1.035 ± 1.179	< 0.001

Values are presented as mean ± standard deviation.

### Global motion

**Fig 2:**
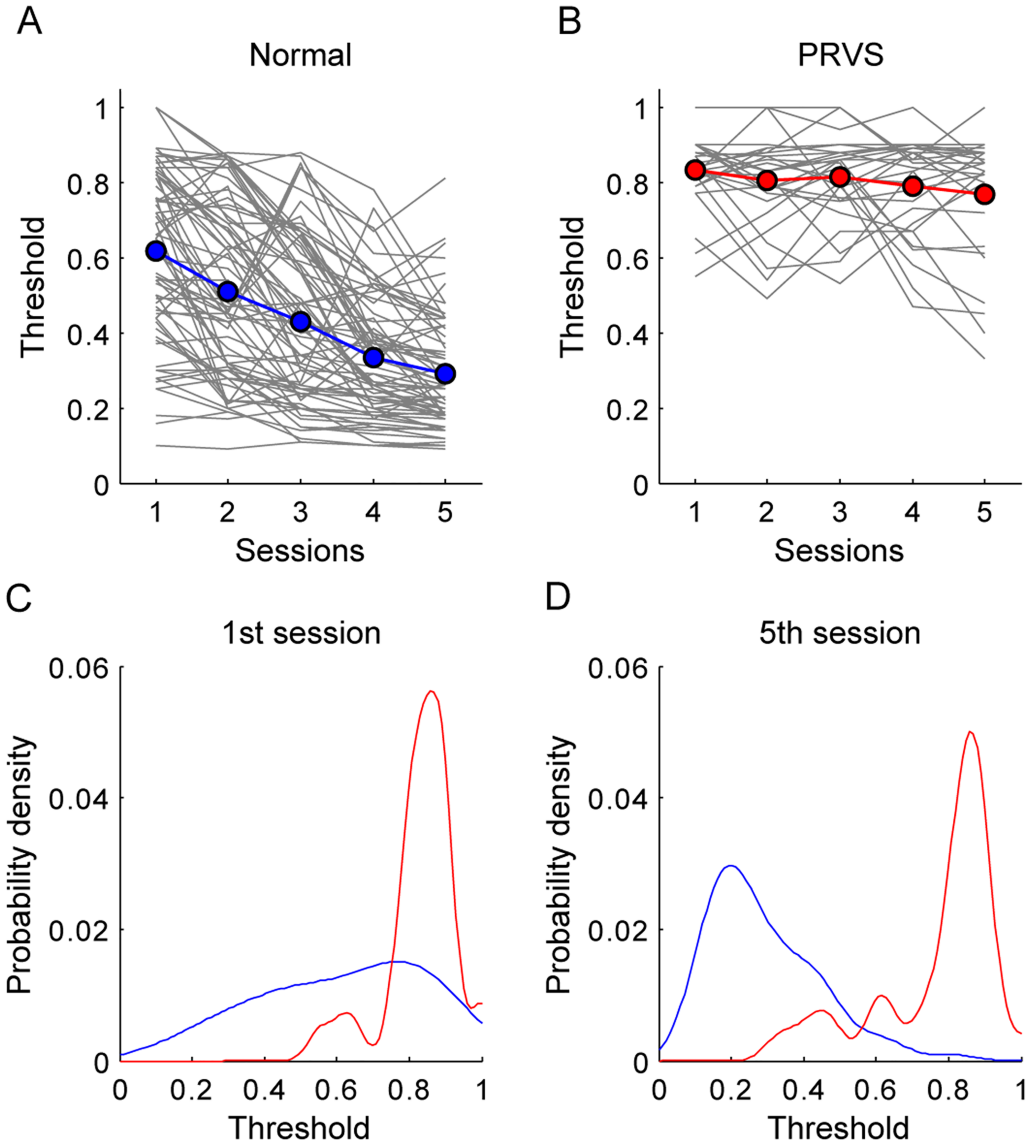
The thresholds to detect global motion reduced after practice. Top panels: Line plot illustrating the changes in threshold to detect global motion with training sessions in normal subjects (A) and those with PRVS (B). Gray lines represent individual subjects’ data and colored symbols represent mean values after each session. Bottom panel: probability density plot for the threshold to detect global motion after the 1^st^ session (C) and the 5^th^ session (D). Blue: normal subjects; red: subjects with PRVS.

All subjects were tested for the threshold to detect global motion 5 times. The baseline threshold, the threshold measured during the first session, in the subjects with PRVS was significantly higher than that of the normal subjects (Fig 2 and Table 2). However, the difference was only about 0.22. Over the sessions, a decrease in the threshold was apparent in the normal subjects. The improvement ratio (IR) was calculated as the (Threshold 1^st^ session—Threshold 5^th^ session)/(Threshold 1^st^ session). The mean IR in the normal was 0.483 ± 0.279, with 87.5% (63/72) subjects having IR > 0.2, and 80.6% (58/72) subjects having IR > 0.3. In contrast, the decrease of threshold was much smaller in the subjects with PRVS. The mean IR was 0.075 ± 0.207 (p < 0.001), with only 24.14% (7/29) subjects having IR > 0.2, and 10.34% (3/29) subjects having IR > 0.3. After 5 sessions of tests, the threshold in the subjects with PRVS was significantly higher than that in the normal subjects (Table 2). The difference became much larger (0.47), instead of disappearing.

**Table 2:** The thresholds to detect global motion after each session.

Sessions	Normal	PRVS	U value	P value (2 tailed)
1st	0.617 ± 0.228	0.832 ± 0.098	411	< 0.001
2nd	0.511 ± 0.229 *	0.806 ± 0.127	297	< 0.001
3rd	0.429 ± 0.219 *	0.814 ± 0.113	146	< 0.001
4th	0.333 ±0.163 *	0.789 ± 0.132	53	< 0.001
5^th^	0.291 ± 0.149 *	0.767 ± 0.170	71.5	< 0.001
A month later	0.407 ± 0.216 *	0.843 ± 0.169	156.5	< 0.001

Values are presented as mean ± standard deviation.

* Indicates a significant difference from the threshold obtained from the 1^st^ session.

**Fig 3:**
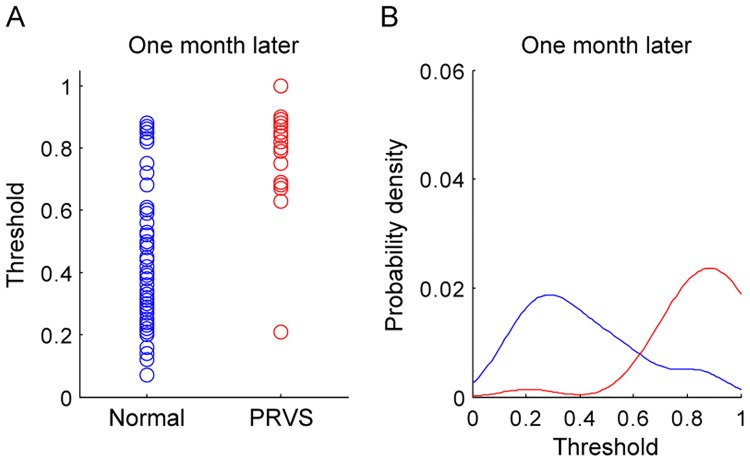
Threshold to detect global motion measured after a break for a month.

To test if the increased difference between normal and PRVS subjects persists after a period without training, the threshold to detect global motion was evaluated again after giving each subject a break for a month (Fig 3). For the normal subjects, the threshold rebounded to a level similar to the third training session (p = 0.542), which was significantly lower than the baseline (p < 0.001). In other word, the effect of practice was partially retained in non-PVRS subjects. In the subjects with PRVS, the threshold rebounded to a level similar to the baseline (p = 0.485). The difference (0.44) between the normal subjects and subjects with PRVS, after a break for a month, was still larger than the difference found at the baseline (0.22).

### Agreement between pattern glare and global motion

**Fig 4:**
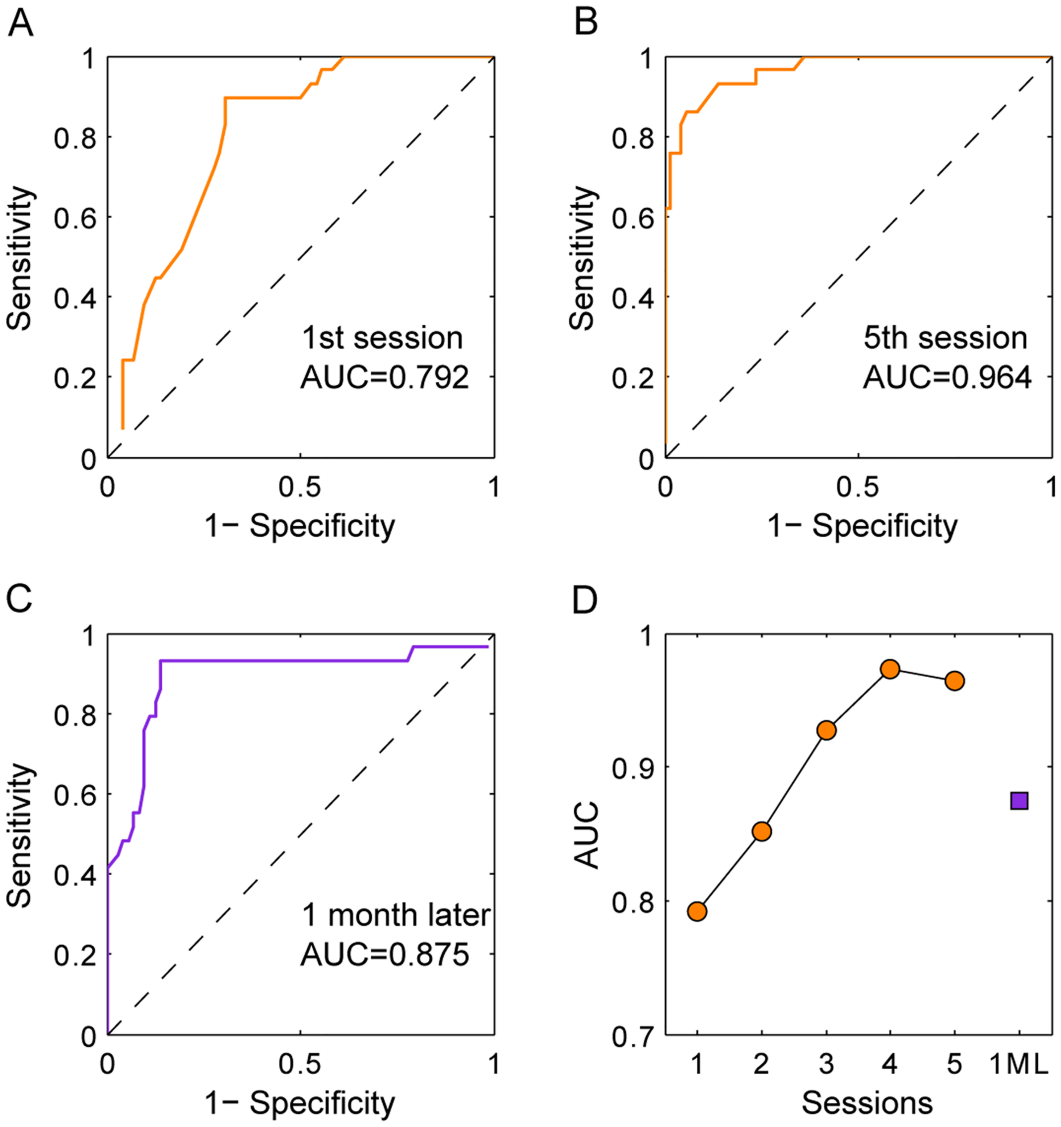
ROC analysis showing agreement between global motion test and pattern glare test. ROC curves after the first session (A), the 5^th^ session (B), and after a break for a month (C). The AUC was plotted as a function of numbers of sessions (D). Orange, sessions in the first visit; purple, after a 1-month break.

ROC analysis was performed after each session to investigate the agreement between the pattern glare test and the global motion test in distinguishing subjects with PRVS from the normal subjects (Fig 4). With a chosen criterion for global motion threshold, a subject with a higher threshold was classified as having PRVS and a subject with a lower threshold was classified as normal. This result was compared to the results from the pattern glare test to calculate the sensitivity and specificity. As the criterion was systemically varied, a ROC curve was constructed and the AUC was calculated as 0.792 at baseline and it improved over the sessions. After 4 sessions of training, it reached a value of 0.973 and then further practice did not improve it. The ROC analysis after the 5^th^ session suggested an optimal cut-off criterion and a threshold of 0.6 of coherence to detect the global motion, with 94.4% specificity and 86.1% sensitivity. Due to the rebound of threshold in both normal and subjects with PRVS, the AUC dropped to 0.875, which as comparable to the AUC value after the 2nd session (0.852) in the first visit.

## Discussion

Our results demonstrated that, at baseline, the subjects with PRVS had a higher threshold than normal subjects in detecting global motion. For most of the normal subjects, but for only some of the subjects with PRVS, the thresholds decreased over sessions of practice. The difference between normal and PRVS subjects became much larger after 5 sessions of practice. The results distinguish PRVS subjects from the normal ones and match well with results from pattern glare test, particularly after practice, with an AUC of 0.964 after 5 sessions.

### Comparison to previous studies

The results from previous studies [34, 35] and current study form a continuum on the performance of detecting in global motion with PRVS. At one extreme, Simmers et al reported no difference between subjects with RPVS and normal subjects. At the opposite extreme, our revealed showed persistence impairment in performance even after five sessions of practice. In the middle, Conlon’s results showed significantly worsen performance that disappeared after second test. The difference among those three studies could be partially be explained by the following factors.

First, the criteria used to establish subjects with PRVS are different. In Simmer’s study, subjects who voluntarily used the color overlay over 6 months were recruited. In Conlon’s study, visual discomfort was assessed with the combination of two methods. One is the Visual Discomfort Scale [39], which measures the retrospective reports on visual discomfort. The other one is a 3-points rating scale to rate the immediate somatic and perceptual unpleasantness of a horizontal square-wave grating with spatial frequency at 4-cycles/degree, without the control stimuli at low and high spatial frequencies. Subjects score 50% or greater on both measures are classified as visual discomfort. In our study, the responses to three spatial frequencies were collected [26].

Second, in our study and Simmer’s study, only one field of dots were used and subjects were required to detect the direction of the coherent motion. In Conlon’s study, two panels each with 300 white dots were presented simultaneously, with one containing variable percentage of dots moving coherently and the other one containing randomly moving dots only. The subject’s task was to select the panel that containing the coherent motion. With the simultaneous presence of the target panel and the reference panel, it becomes a discrimination task, instead of detection task that is harder. This might explain the observed large improvement of threshold in subjects with visual discomfort.

Third, in Conlon’s study, the subjects were only tested twice on two sessions. As shown in our study, even after the second session of tests, there was still some overlap between the normal subjects and the PRVS subjects since the AUC was 0.852. Considering with the relative smaller sample size in Conlon’s study (n = 17 for normal subjects with visual discomfort), it is highly possible to get a result showing no statistical difference.

Fourth, the parameter values used for global motion tests were different in those studies. It is known that parameters including dots numbers, dots density, dots luminance, moving speed, etc., could influence the measured threshold [40, 41]. In Simmer’s test, both black and white dots were used. There are fewer dots (100 vs. 200 in ours) moving at lower speed (2.5 deg/sec vs. 5 deg/sec in ours). The dots subtended over smaller circular space (4-degree vs. 12-degree in ours). Each dot in Simmers’ study is smaller (0.03 deg vs. 0.16 deg in ours) and has shorter lifetime (26 ms vs. 200 ms in ours). It is possible that visual stimulus in our study could have triggered PRVS in the sensitive subjects, while the stimuli used in Simmers’ study did not. With or without color overlay, the subjects’ performance in Simmers’ study did not show any significant difference, perhaps supporting the notion that Simmers et al.’s motion test did not elicit symptoms of visual stress but our did.

### Close association between the subjective test and objective test

It is important to note the close agreement between the pattern glare test and global motion test in distinguishing subjects with PRVS from the normal subjects. Pattern glare is based on an individuals’ subjective report of perceived symptoms [26]. Our global motion test is a 2-alternative forced choice test that minimizes the influence of subjective bias of individual subjects. Significantly in our study, objective results matched results from the subjective assessment. This close association could be due to underlying neural mechanism. The prevailing explanation for PRVS is the cortical hyperexcitability [14, 42]. In normal subjects, it has been proved that, for noise-free tasks, increased cortical excitability by external stimulation improves the performance and decreased cortical excitability deteriorates the performance [28]. More importantly, for tasks with noise, reduction of the cortical excitability actually enhances the performance. In PRVS, a stimulus-driven cortical hyperexcitability could impair one’s capability to separate the noise from signal. According to these, the altered motion perception in subjects with PRVS would be more reasonably interpreted as the effect of cortical hyperexcitability, rather than the cause.

Global motion is processed in the dorsal pathway, especially the V5 area where the neurons are shown to have larger receptive fields [43, 44]. By using the global motion, our study and others suggested that PRVS affects visual processing outside the primary visual cortex [35]. It also suggested repetition affects visual tasks in the extrastriate areas differently in the people with PRVS than the normal populations. Further study is desirable to better understand the effects of repetitive visual processing tasks in the subjects who are deficient in inhibitory suppression such as migraine and PRVS. This type of visual stimulus is of greater significance as daily tasks increasing consist of high contrast computer generated images.

One precaution that we should keep in mind is that, even after 5 sessions of training, the sensitivity to distinguish subjects, with a cut off threshold of 60% coherence to detect global motion, was still only 86.1%, not quite as high as the specificity. In other words, there is a partial overlap between the normal population and the population with PRVS. Or it could simply be that global motion processing and PRVS share certain portions of the neural mechanism, not the entire neural underpinnings.

### Some other noticeable points in this study

Several previous studies on perceptual learning reported that the subjects with worse performance at the start of training tend to improve much more than those with better performance [45, 46]. That was the case for the normal subjects in our study, but was not true for the subjects with PRVS. The improvement ratio in subjects with PRVS was significantly smaller than that in normal subjects. It is possible that, in subjects with PRVS, repetitive exposure to the visual stimuli would further increase the cortical excitability, which in turn would counter act the learning effects from practice and lead to small or no improvement at all.

Some previous studies on perceptual learning have tried to equate the performance levels before training by scaling the stimuli to see if that could lead to an equivalent amount of improvement throughout the training [47]. In this study, we purposely used subjects who are naïve to psychophysical experiment to remove the potential interference of previous learning experience. This allowed us to better reveal the differences of the initial condition between the normal subjects and subjects who are sensitive to pattern glare. Every subject started the test with a 100% coherence level and followed the same 3-down-1-up procedure. We did not adjust the initial values in the following session.

It would be interesting to determine how long the positive effect of perceptual learning could last. In our study, we did not intentionally test the lasting duration. We simply noticed that in the evaluation 1 month after the first 5 sessions of training, the subjects’ performance showed a significant regression. However, they were still significantly better than the values obtained at the first measurement, which mean the learned effect was retained for at least 1 month. For the subjects who were sensitive to pattern glare, the rebound effect was not as dramatic as in the normal subjects. This does not mean that the subjects who were sensitive to pattern glare retained the learned function better; it was simply due to the fact that the reduction in threshold from the training sessions that occurred a month ago were not as great as those in normal subjects to begin with. In other words, there was not much space for rebounding.

### The limitations of current study

We compared our normal subjects with other studies to ensure that they are indeed normal. For PGT test, the scores to gratings at all three spatial frequencies were within the normal ranges established by Evans et al. The mean scores to gratings at low, middle, and high spatial frequency were 0.52, 1.72, and 2.11 respectively, which were very close to the normative values reported by Evans et al. (0.53, 1.59, and 1.82) [26]. Moreover, the difference between scores to middle and high spatial frequency was -0.39 in our study and -0.23 in Evans’ study. For global motion, the normal subjects mean thresholds (0.29 ± 0.15) were very close to those reported in the studies with similar choice of parameters (0.37 ± 0.10) [28].

However, caution has to be applied that when interpret the findings on the subjects with visual stress. From above comparison, it is clear that when the diagnosis of visual stress relies on either symptoms or signs, the findings may vary. When this study was already in the data collection stage, no unified diagnostic criteria with balanced utilization of signs and symptoms were available. Fortunately, in two recently published studies [48, 49], Evans and coworkers have filled this gap. They identified the six most important symptoms include words moving, merging, and fading, patterns and shadows in text, text standing above page, and discomfort to flickers. They also pointed out the three most important signs include voluntary use of a color overlay over 3 month, an improvement more than 15% in Wilkins Rate of Reading Test when using colored filters, PGT result greater than 3 with mid-spatial frequencies. It is recommended that at least three of the six typical symptoms and two of the three signs should be present for a visual stress diagnosis. The emergence of such diagnostic standard will greatly push forward the research in this field. Another point that we want to point out is that we could not exclude the influence about the potentially co-existing conditions, such as certain learning difficulties, migraine, epilepsy, and medications on the global motion results.

## Conclusions

Subjects with PRVS are less sensitive at detecting global motion. This difference becomes significantly greater, instead of disappearing, over sessions of practice.
